# U-Shaped Split Federated Learning with Compact Features for Deep Learning-Based Image Coding

**DOI:** 10.3390/e28030331

**Published:** 2026-03-16

**Authors:** Qizheng Sun, Caili Guo, Meiyi Zhu, Yang Yang

**Affiliations:** 1 Beijing Key Laboratory of Network System Architecture and Convergence, School of Information and Communication Engineering, Beijing University of Posts and Telecommunications, Beijing 100876, China; qizheng_sun@bupt.edu.cn (Q.S.); yangyang01@bupt.edu.cn (Y.Y.); 2 Department of Engineering, King’s College London, London WC2R 2LS, UK; meiyi.1.zhu@kcl.ac.uk

**Keywords:** U-shape split federated learning, image coding, distributed learning, entropy estimation, joint source channel coding

## Abstract

U-shaped Split Federated Learning (U-SFL) is a promising paradigm for distributed image coding, offering parallel training capabilities and privacy preservation while mitigating computational burdens on edge devices. However, the frequent bidirectional transmission of intermediate features between dual-split points incurs substantial communication overhead. To mitigate this issue, we propose a compact-feature U-shaped split federated learning framework (CoF U-SFL), which reduces communication overhead and improves training efficiency while maintaining low image distortion. We introduce a feature entropy estimation network to model the distribution of split-layer features, enabling effective compression during transmission. Furthermore, we formulate a joint optimization objective incorporating entropy constraints to guide the end-to-end training. Experimental results demonstrate that CoF U-SFL reduces communication overhead by 104.6 times while maintaining reconstruction performance.

## 1. Introduction

With the exponential growth of image data, a vast amount of visual information is continuously generated at edge devices, necessitating efficient image coding methods to support effective transmission and storage. Traditional approaches typically rely on centralized learning (CL) frameworks, where raw image data from all devices is transmitted to a central server for unified model training. However, this paradigm faces significant uplink communication overhead and poses severe privacy leakage risks, making it challenging to deploy in practical and data-sensitive scenarios [1].

To address privacy concerns, federated learning (FL) [2,3,4] has been proposed. In FL, clients train the entire model locally and only upload model parameters, thereby avoiding the exposure of raw data. However, FL implicitly assumes that edge devices have the capability to deploy and execute large-scale deep neural networks, especially those used for high-fidelity image encoding and decoding, such as autoencoders or Transformers. This assumption is often invalid in resource-constrained edge devices due to their strict limitations in computational power and memory resources.

To alleviate these limitations, split learning (SL) [5] was introduced by partitioning the model at specific layers: only the initial shallow layers are deployed on the client side, while the computationally intensive deeper layers are offloaded to the server. The training process involves iteratively exchanging intermediate activations and gradients between the split layers. Although this approach significantly reduces the computational and memory requirements on the client’s side, it introduces considerable time delays and significant communication overhead due to the frequent transmission of high-dimensional intermediate data.

Recent studies have introduced split federated learning (SFL) [6,7,8,9] to combine the strengths of FL and SL. SFL retains the parallel training capability of FL while employing model partitioning to reduce the computational burden on clients. Similar to SL, SFL also suffers from significant communication overhead due to the frequent exchange of high-dimensional intermediate activations and gradients. Furthermore, SFL faces a privacy challenge in image coding tasks: calculating reconstruction loss requires access to original images, which cannot be transmitted to the server.

To enable collaborative training while preserving data confidentiality in image coding tasks, the U-shaped split federated learning (U-SFL) framework [10,11,12,13] was proposed. This framework partitions the global model into a client-side head, a server-side body, and a client-side tail network. Consequently, original images remain local, and optimization relies solely on the bidirectional exchange of intermediate data. U-SFL has demonstrated its effectiveness in preserving data privacy across various applications, including medical imaging [12], vehicular networks [11], and facial privacy protection [13]. However, the dual-split architecture necessitates the transmission of two sets of high-dimensional tensors, namely forward activations and backward gradients, during each training round. This results in significantly higher communication overhead than single-split SL or SFL, creating a bottleneck for training efficiency in bandwidth-constrained edge environments.

To alleviate this burden, various compression techniques have been developed for distributed learning paradigms. In FL, representative approaches include parameter coding via Gaussian mixture models [14], clustering-based dimensionality reduction [15], autoencoder-assisted model update compression and reconstruction [16], and attention-driven adaptive fusion of client updates [17]. Additionally, recent advances explore methods like sparse subnetwork identification through weight freezing and binary mask transmission [18]. In SL, compression targets intermediate representations directly. Notable methods include end-to-end feature compression using autoencoders [19], batch-level activation fusion through cyclic convolutions in C3-SL [20], and channel-wise sparsification based on adaptive importance scoring in SL-ACC [21]. In SFL, further innovations include (i) local loss-driven parallel training to eliminate server-to-client communication [22]; (ii) auxiliary networks enabling client-side weight updates to reduce synchronization frequency [23]; and (iii) quantization-based compression techniques, ranging from system-aware dynamic model splitting for latency reduction [24] to adaptive feature-wise dropout and quantization for bandwidth efficiency [25]. However, these compression approaches often incur distortion in the transmitted features. In contrast, compression research for U-SFL is still in its early stages. Existing efforts [11] primarily rely on simplifying the client head; for example, by fixing its parameters or employing fixed embedding modules. Although semantic autoencoders (SAEs) have been explored to reduce feature dimensionality [26], such projection-based compression often introduces distortions that degrade reconstruction fidelity. Consequently, there remains a need for an efficient, low-distortion compression mechanism specifically designed for the bidirectional high-dimensional data streams in U-SFL.

In this paper, we propose a U-shaped split federated learning framework with compact features (CoF U-SFL), which aims to reduce communication overhead and improve training efficiency while maintaining low image distortion and strong privacy protection. The main contributions of this paper are summarized as follows:

- We propose a feature entropy estimation network designed to model the statistical distribution of split-layer features. By utilizing a dual-layer Transformer-based hyperprior architecture, this network achieves accurate entropy estimation without accessing raw data, achieving a favorable balance between estimation accuracy and model complexity.
- We introduce CoF U-SFL, a novel U-shaped split federated learning framework that supports compact feature representations. By leveraging an entropy-guided quantization strategy, our method significantly reduces the bit-width of high-dimensional intermediate features while preserving image reconstruction quality and inherently guaranteeing client-side data privacy.
- We establish a robust training pipeline governed by a joint objective function that integrates entropy constraints with image distortion metrics. This formulation enables unified optimization of both the entropy estimation network and the image coding network, facilitating convergence towards optimal coding efficiency.
- We conduct comprehensive experiments across multiple datasets to validate the effectiveness of CoF U-SFL. Results demonstrate that the proposed method reduces communication overhead by approximately 104.6 times, while maintaining comparable image reconstruction performance.

## 2. Related Work

### 2.1. Evolution of Collaborative Learning Paradigms

Collaborative learning has evolved through several paradigms to address the tension between data privacy and computational constraints. Initially, FL [2,3,4] was introduced to keep raw data local. However, the requirement for clients to execute full models imposes substantial computational burdens. To mitigate this, SL [5,27] partitions the model, offloading intensive deep layers to a server. Building on this, SFL [6,7,8,9] incorporates parallelism into SL to improve scalability. For tasks specifically requiring end-to-end data confidentiality, such as image coding, U-SFL [10,11] has emerged as the key architecture. Yang et al. [12] applied it to medical image restoration for patient data protection, Zheng et al. [11] utilized it in vehicular perception networks, and other studies have extended it to facial privacy preservation [13]. The evolution of collaborative learning has been driven by the dual need for data privacy and resource efficiency, with communication compression playing a pivotal role in each paradigm [24], particularly for handling the communication-intensive bidirectional transmission characteristic of U-SFL.

### 2.2. Communication Compression in Federated Learning

In FL, communication bottlenecks arise from the transmission of high-dimensional model updates. To address this, statistical approaches have been proposed, such as encoding gradients via Gaussian mixture models [14] or employing clustering-based dimensionality reduction [15]. Structural optimization methods further reduce overhead through sparse subnetwork identification using binary masks [18] or attention-driven adaptive fusion [17]. Additionally, auxiliary-based techniques utilize autoencoders to compress and reconstruct model updates [16]. However, these methods focus exclusively on compressing static model parameters or gradients derived from full-model local training. They are inapplicable to split architectures, where the communication payload consists of dynamic intermediate features that vary with each input sample.

### 2.3. Intermediate Feature Compression in Split Learning

In SL, the frequent transmission of high-dimensional intermediate activations constitutes a major bottleneck. Compression techniques have been developed to target these representations directly. End-to-end approaches integrate lightweight autoencoders at the split layer to compress features [19]. Batch-level strategies, such as C3-SL [20], employ cyclic convolutions to fuse activation maps from multiple samples into a compact representation. Furthermore, structural sparsification methods, like SL-ACC [21], utilize adaptive importance scoring to transmit only task-sensitive channels. However, these methods are inherently designed for a single split point. They do not account for the dual-split architecture of U-SFL, where error signals need to propagate through two independent network segments. Compared to standard SL, U-SFL involves more complex bidirectional interactions among the head, body, and tail networks.

### 2.4. Compression Mechanisms in Split Federated Learning

SFL combines the parallelism of FL with the model partitioning of SL, necessitating strategies that simultaneously handle aggregation and feature transmission. Communication path elimination has been proposed to enable local loss-driven parallel training, effectively removing server-to-client gradient transmission [22]. To reduce synchronization frequency, auxiliary networks have been introduced to approximate server-side gradients locally [23]. Recent works have also explored quantization-based techniques, ranging from system-aware dynamic model splitting [24] to adaptive feature-wise dropout combined with quantization [25]. Despite these advances, quantization and dropout-based methods inevitably introduce information distortion in the transmitted features. Furthermore, methods relying on local losses to eliminate the backward path [22] are structurally incompatible with U-SFL. Since U-SFL computes the loss at the client-side tail, a complete backward communication link is strictly required to propagate supervision signals back to the body and head networks.

### 2.5. Compression Challenges and Research Gap in U-SFL

U-SFL achieves end-to-end privacy preservation by placing both the encoder head and decoder tail on the client, effectively keeping raw inputs and reconstruction targets local. However, this architectural benefit comes at the cost of doubled communication overhead compared to standard SL, as it requires the bidirectional exchange of high-dimensional intermediate features. Currently, compression research tailored for U-SFL remains nascent. Existing implementations, such as those by Zheng et al. [11], primarily simplify the client head by fixing its parameters or utilizing non-trainable embedding modules, thereby avoiding explicit feature compression at the expense of model flexibility. While recent attempts have employed Semantic Autoencoders (SAEs) to reduce feature dimensionality [26], such projection-based methods often introduce uncontrollable distortion. In summary, the current literature lacks a unified framework that simultaneously addresses the bidirectional communication bottleneck and the high-fidelity reconstruction requirement of U-SFL.

## 3. System Model

**Figure 1:**
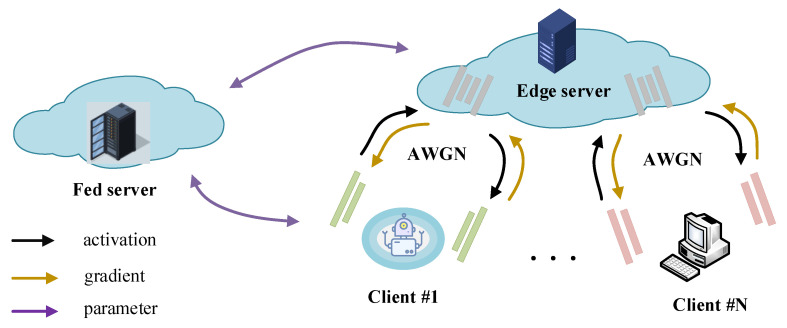
The system model of the proposed CoF U-SFL method.

This section establishes the system model for the proposed CoF U-SFL scheme. As illustrated in Figure 1, the system primarily consists of *N* distributed clients (denoted by the set N={1,…,N}), an edge server, and a Federated (Fed) server. During distributed training, the edge server and the clients collaboratively execute the forward and backward propagation processes. Specifically, the edge server hosts the intermediate body network, while the clients manage their respective head and tail networks. Crucially, to prevent reconstruction attacks arising from simultaneous access to models and activations [28], the Fed server operates independently from the edge server to perform periodic model aggregation.

In the end-to-end deep joint source channel coding (JSCC) pipeline, let $x_{i}$ denote the raw input data of client *i*. To account for the stochastic nature of wireless transmission environments within the U-SFL architecture, the feature exchange process between the client and the edge server is modeled as an additive white Gaussian noise channel (AWGN) at the two split points involving the head–body interface and the body–tail interface. The received feature $a_{i}^{\prime }$ can be expressed as

$$a_{i}^{\prime }=a_{i}+n,$$

where $a_{i}$ represents the original feature representation transmitted at the corresponding split point, and $n∼N(0,\sigma^{2}I)$ denotes the noise term introduced by the AWGN channel. The communication quality of this link is quantified by the signal-to-noise ratio (SNR) predefined during training.

During the model optimization phase, the AWGN channel is directly embedded into the end-to-end computational graph, effectively serving as a non-trainable stochastic linear operator. In the forward pass, the system performs noise sampling and overlays it onto the features at each split point, ensuring that subsequent networks operate on the received features $a_{i}^{\prime }$ to compute the training loss. Under this mechanism, the channel noise participates in the closed-loop gradient conduction via the chain rule, thereby imposing the stochastic constraints of the communication environment directly onto the parameter updates of each sub-network. This channel-aware training mechanism [29] drives the model to extract channel-robust features, implicitly incorporating error tolerance into the training process. Furthermore, following the classical assumption in deep JSCC [29], this paper assumes error-free gradient transmission during back-propagation to ensure the convergence stability of the distributed model training.

Since U-SFL requires frequent exchange of features $a_{i}$ at both split points during training, the communication overhead becomes a core bottleneck. Therefore, it is necessary to investigate methods for reducing the feature data volume during training while maintaining image quality.

## 4. The Proposed U-Shaped Split Federated Learning Framework with Compact Features

In this section, we elaborate on the proposed U-shaped split federated learning framework with compact features (CoF U-SFL). Specifically, this framework aims to reduce communication overhead and improve efficiency during the collaborative training process, while maintaining low image distortion and strong privacy protection. We first present the overall system architecture and the bidirectional data flow in Section 4.1. Subsequently, we detail the core component of our method, the Transformer-based feature entropy estimation module, which captures the statistical distribution of intermediate features for accurate bit-rate estimation in Section 4.2. Then, the joint optimization objective, which balances the communication rate of intermediate features and image reconstruction fidelity, is formulated in Section 4.3. Finally, we describe the end-to-end collaborative training procedure in Section 4.4.

### 4.1. Framework Overview

**Figure 2:**
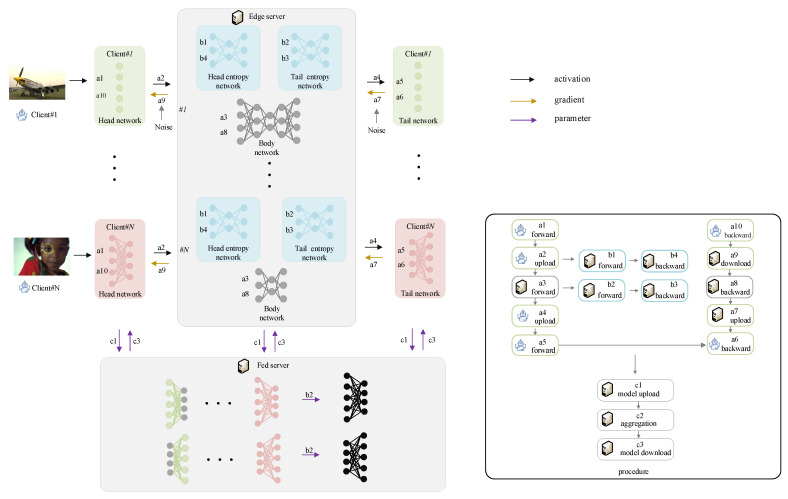
The detailed workflow of the proposed CoF U-SFL framework: (a) Forward and backward propagation of the primary image coding model (a1–a5 for forward propagation, a6–a10 for backward propagation); (b) Forward and backward propagation of the entropy estimation model for feature compression; (c) Global parameter aggregation via the Fed server.

As depicted in Figure 2, each client *i* processes its local dataset $D_{i}$ using the resident head network $w_{ch,i}$ and tail network $w_{ct,i}$. The heavy computational load is offloaded to the edge server, which executes the body network $w_{sb,i}=\left[\theta_{sb};\theta_{m,i}\right]$. To handle client heterogeneity, this body network comprises a shared part $\theta_{sb}$, synchronized per round, and a personalized part $\theta_{m,i}$, which is aggregated every *I* rounds.

This architecture establishes an end-to-end JSCC pipeline [30,31] between clients and the edge server. To minimize communication overhead, Transformer-based entropy estimation modules are embedded at the split points to guide efficient feature compression (detailed in Section 4.2). Furthermore, the entire system is driven by a joint optimization objective that balances reconstruction fidelity and transmission rate (formulated in Section 4.3). In Figure 2, data flows are color-coded, where black, yellow, and purple lines represent the transmission of features, gradients, and parameters, respectively.

### 4.2. Transformer-Based Feature Entropy Estimation Mechanism

In this subsection, we propose a specialized feature entropy estimation mechanism to capture the spatial redundancy within the split-layer features.

**Figure 3:**
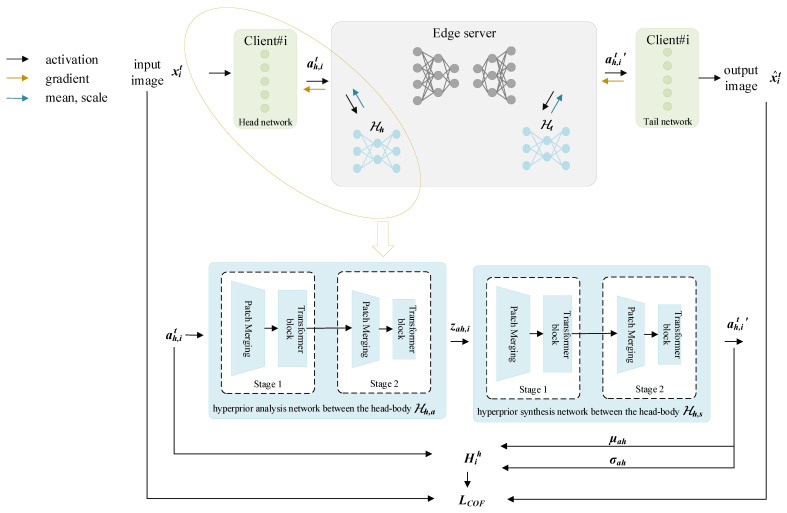
Detailed illustration of the feature entropy estimation process at the split point. The figure depicts the internal structures of the hyperprior networks, demonstrating how these modules estimate the spatial mean and scale parameters to formulate the training loss. Specifically, the black, yellow, and blue arrows indicate the forward activations, backward gradients, and the estimated parameters ($\mu_{ah}$ and $\sigma_{ah}$), respectively.

As illustrated in Figure 3, the mechanism comprises two distinct modules: the head–body entropy estimation network and the body–tail entropy estimation network. Both modules incorporate a dual-layer Transformer-based hyperprior architecture, utilizing self-attention mechanisms to effectively model the global dependencies of the feature distribution. Regarding the architectural depth, we specifically adopt a dual-layer configuration to balance performance and overhead. A single-layer structure often lacks the representational capacity to accurately estimate the distribution parameters, while a deeper network inevitably increases computational latency. Therefore, the proposed dual-layer design aims to ensure sufficient estimation fidelity while maintaining high computational efficiency. This module incorporates hyperprior analysis and synthesis networks (comprising patch merging and transformer blocks) to estimate the spatial mean (μ) and scale (σ) of the intermediate features. This estimated distribution is subsequently utilized to calculate the feature entropy and integrated into the final loss function, guiding the end-to-end rate-distortion optimization.

The detailed workflow begins at the head–body interface. First, the raw image is processed by the head network to generate intermediate features $a_{h,i}^{t}$:

(1) $$a_{h,i}^{t}=H\left(x_{i}^{t};w_{ch,i}^{t-1}\right),$$

where H(·) denotes the mapping function of the head network, and $w_{ch,i}^{t-1}$ represents its learnable parameters. To estimate the bit-rate, we employ a hyperprior network consisting of an analysis network and a synthesis network. Specifically, $a_{h,i}^{t}$ is compressed by the hyperprior analysis network to obtain the latent representation $z_{ah,i}^{t}$:

(2) $$z_{ah,i}^{t}=H_{h,a}\left(a_{h,i}^{t};{\mathrm{ha}}_{sh,i}^{t-1}\right),$$

where $H_{h,a}\left(\cdot \right)$ consists of two stacked Transformer layers capturing global context, and ${\mathrm{ha}}_{sh,i}^{t-1}$ denotes the parameters of the hyperprior analysis network. Subsequently, $z_{ah,i}^{t}$ is decoded by the hyperprior synthesis network to predict the parameters of the underlying Gaussian distribution, yielding the mean $\mu_{ah}$ and variance $\sigma_{ah}$:

(3) $$\mu_{ah},\sigma_{ah}=H_{h,s}\left(z_{ah,i}^{t};{\mathrm{hs}}_{sh,i}^{t-1}\right),$$

where $H_{h,s}\left(\cdot \right)$ denotes the mapping function of hyperprior synthesis network, and ${\mathrm{hs}}_{sh,i}^{t-1}$ represents the parameters of the synthesis network.

Through this process, the distribution of the features at the head–body interface is estimated, enabling the computation of the Shannon cross-entropy [32] as follows:

(4) $$H_{i}^{h}=E_{x_{i}∼p_{x_{i}}}\left[-\log_{2}\;p_{a_{h,i}^{t}}\left(a_{h,i}^{t}|\mu_{ah},\sigma_{ah}\right)\right],$$

where $p_{a_{h,i}^{t}}$ denotes the probability model predicted by the hyperprior network at the head–body interface, and $E_{x_{i}∼p_{x_{i}}}\left[\cdot \right]$ represents the expectation over the input data distribution.

Similarly, at the body–tail interface, the entropy estimation mechanism with an identical architecture is applied. Specifically, the features at the body–tail interface $a_{t,i}^{t}$ are compressed into a latent representation $z_{at,i}^{t}$, and subsequently estimate their distribution parameters. The corresponding formulations are given by

(5) $$\begin{array}{cc} z_{at,i}^{t} & =H_{t,a}\left(a_{t,i}^{t};{\mathrm{ha}}_{st,i}^{t-1}\right), \end{array}$$

(6) $$\begin{array}{cc} \mu_{at},\sigma_{at} & =H_{t,s}\left(z_{at,i}^{t};{\mathrm{hs}}_{st,i}^{t-1}\right), \end{array}$$

where $H_{t,a}\left(\cdot \right)$ and $H_{t,s}\left(\cdot \right)$ denote the hyperprior analysis and synthesis network parameterized by ${\mathrm{ha}}_{st,i}^{t-1}$ and ${\mathrm{hs}}_{st,i}^{t-1}$, respectively. Consequently, the entropy at the body–tail interface is derived as

(7) $$H_{i}^{t}=E_{x_{i}∼p_{x_{i}}}\left[-\log_{2}\;p_{a_{t,i}^{t}}\left(a_{t,i}^{t}|\mu_{at},\sigma_{at}\right)\right],$$

where $p_{a_{t,i}^{t}}$ represents the probability model predicted by the hyperprior network at the body–tail interface.

### 4.3. Joint Optimization Objective

To achieve high-performance distributed learning under bandwidth constraints, the CoF U-SFL framework is trained using a joint rate-distortion optimization strategy. The objective function is designed to minimize the system’s end-to-end distortion while strictly constraining the entropy of the intermediate features transmitted over the wireless channel.

Formally, the total loss function of proposed CoF U-SFL $L_{\mathrm{CoF}}$ is formulated as a weighted sum of the distortion loss and the entropy estimates:

(8) $$L_{\mathrm{CoF}}=\underset{\mathrm{Distortion}}{\underbrace{d(x,x^{\prime })}}+\underset{\mathrm{Rate}}{\underbrace{\lambda_{h}H_{i}^{h}+\lambda_{t}H_{i}^{t}}},$$

where $d(x,x^{\prime })$ denotes the mean squared error (MSE) quantifying the reconstruction loss between the input x and the output $x^{\prime }$. The terms $H_{i}^{h}$ and $H_{i}^{t}$ represent the cross-entropy of the feature distributions at the head–body and body–tail interfaces, respectively. The coefficients $\lambda_{h}$ and $\lambda_{t}$ serve as Lagrange multipliers that regulate the trade-off between reconstruction fidelity and communication efficiency.

This formulation effectively compels the U-SFL networks to learn compact feature representations with low spatial redundancy, thereby minimizing the transmission latency without compromising the overall model performance.

It is worth noting that directly optimizing the joint loss function $L_{\mathrm{CoF}}$ from the beginning of training may lead to instability, as the network struggles to balance reconstruction quality and entropy constraints simultaneously. To mitigate this, we adopt a two-stage training strategy. In the warm-up phase, we set $\lambda_{h}=\lambda_{t}=0$ and optimize the network solely using the distortion loss $d(x,x^{\prime })$. This allows the head, body, and tail networks to establish a stable feature representation and achieve a baseline reconstruction quality. Once the distortion loss stabilizes, we transition to the second phase by activating the entropy constraints by setting $\lambda_{h},\lambda_{t}>0$ to optimize the full objective $L_{\mathrm{CoF}}$. This strategy ensures robust convergence and prevents the model from collapsing into low-quality solutions driven by excessive rate penalties.

### 4.4. Training Procedure

The training procedure of the proposed CoF U-SFL framework is organized into three distinct phases. These phases are (a) *image coding network training*, which handles the end-to-end forward and backward propagation for the head, body, and tail networks; (b) *entropy network training*, which involves the forward and backward propagation for the head and tail entropy networks, responsible for estimating the feature entropy at the head–body and body–tail interfaces, respectively; and (c) *global model aggregation*, performed periodically every *I* rounds to synchronize the head network, tail network, and the personalized part of the body network. Let *t* denote the current training round. The detailed workflow is described as follows.

(a) Image Coding Network Training: In this phase, the client and edge server collaboratively update the image coding parameters ($w_{ch},w_{sb},w_{ct}$) through a sequence of forward propagation (FP) and backward propagation (BP), corresponding to steps a1–a5 and a6–a10 in Figure 2, respectively. Specifically, in training round *t*, client *i* employs the local head network to extract the feature map $a_{h,i}^{t}$ at the head–body interface:

(9) $$a_{h,i}^{t}=H\left(x_{i}^{t};w_{ch,i}^{t-1}\right),$$

where H(·) represents the head network mapping function, and $w_{ch,i}^{t-1}$ denotes the corresponding model parameters from the previous round. Subsequently, the extracted feature map $a_{h,i}^{t}$ is transmitted to the edge server. Modeling the wireless channel as an AWGN channel, the received signal at the server is expressed as

(10) $${a_{h,i}^{t}}^{\prime }=a_{h,i}^{t}+n,$$

where n represents the noise of the channel, with its elements following a distribution $N(0,\sigma^{2})$. Upon reception, the edge server executes the body network FP to extract the feature map $a_{t,i}^{t}$ at the body–tail split point:

(11) $$a_{t,i}^{t}=B\left({a_{h,i}^{t}}^{\prime };w_{sb,i}^{t-1}\right),$$

where B(·) represents the body network mapping, and $w_{sb,i}^{t-1}$ denotes the corresponding body network parameters from the previous round. The server then transmits $a_{t,i}^{t}$ back to the client over the AWGN channel, resulting in the received tail input ${a_{t,i}^{t}}^{\prime }=a_{t,i}^{t}+n$. Finally, the client reconstructs the image via the FP of the local tail network:

(12) $${\hat{x}}_{i}^{t}=T\left({a_{t,i}^{t}}^{\prime };w_{ct,i}^{t-1}\right),$$

where T(·) denotes the tail network mapping, $w_{ct,i}^{t-1}$ denotes the corresponding tail network parameter, and ${\hat{x}}_{i}^{t}$ represents the reconstructed image.

Following the loss calculation, gradients are back-propagated to update the sub-networks in reverse order. First, the client *i* independently updates the tail network parameters $w_{ct,i}$ via gradient descent:

(13) $$w_{ct,i}^{t}\leftarrow w_{ct,i}^{t-1}-\gamma \nabla_{w_{ct}}F_{i}\left(w_{ct,i}^{t-1};\xi_{i}^{t}\right),$$

where γ is the learning rate, and $\nabla_{w}F\left(\cdot \right)$ denotes the parameter gradient. $\xi^{t}≜{\left[\xi_{i}^{\tau }\right]}_{i\in \{1,2,\ldots ,N\},\tau \in \{1,\ldots ,t\}}$ represents all the randomness. The gradients of the activations are then transmitted back to the server. Next, the server updates the body network. As defined in Section 4.1, the body network $w_{sb}$ comprises a shared part $\theta_{sb}$ and a personalized part $\theta_{m,i}$. The *shared part* aggregates gradients from all *N* clients to emulate centralized training:

(14) $$\theta_{sb}^{t}=\frac{1}{N}\sum_{i=1}^{N}\left(\theta_{sb,i}^{t-1}-\gamma \nabla_{\theta_{sb}}F_{i}\left(\theta_{sb,i}^{t-1};\xi_{i}^{t}\right)\right),$$

where $\nabla_{\theta_{sb}}F_{i}\left(\theta_{sb,i}^{t-1};\xi_{i}^{t}\right)$ represents the gradients of the *shared part* of the body network. The *personalized part*, which handles client-specific heterogeneity, is updated independently for each client *i*:

(15) $$\theta_{m,i}^{t}\leftarrow \theta_{m,i}^{t-1}-\gamma \nabla_{\theta_{m}}F_{i}\left(\theta_{m,i}^{t-1};\xi_{i}^{t}\right),$$

where $\nabla_{\theta_{m}}F_{i}\left(\cdot \right)$ represents the gradient with respect to the *personalized part* of body network parameters for client *i*. Finally, the edge server returns the gradients to the clients, enabling the final update of the client-side head network:

(16) $$w_{ch,i}^{t}\leftarrow w_{ch,i}^{t-1}-\gamma \nabla_{w_{ch}}F_{i}\left(w_{ch,i}^{t-1};\xi_{i}^{t}\right).$$

(b) *Entropy network training*: This phase focuses on optimizing the hyperprior networks to accurately estimate the probability distributions of the features. First, the server performs the forward propagation of the entropy estimation network to calculate the head-side entropy $H_{i}^{h}$, as defined in Equations (2)–(4). Subsequently, at the head–body interface, the server updates the parameters of the hyperprior analysis and synthesis networks, denoted as ${\mathrm{ha}}_{sh,i}^{t-1}$ and ${\mathrm{hs}}_{sh,i}^{t-1}$, to minimize the total loss $L_{\mathrm{CoF}}$. This ensures that the probability model $p_{a_{h,i}^{t}}$ closely fits the distribution of the features at the head–body interface. The update rule is formulated as

(17) $$\begin{array}{cc} {\mathrm{ha}}_{sh,i}^{t} & \leftarrow {\mathrm{ha}}_{sh,i}^{t-1}-\eta \nabla_{{\mathrm{ha}}_{sh,i}}L_{\mathrm{CoF}}, \end{array}$$

(18) $$\begin{array}{cc} {\mathrm{hs}}_{sh,i}^{t} & \leftarrow {\mathrm{hs}}_{sh,i}^{t-1}-\eta \nabla_{{\mathrm{hs}}_{sh,i}}L_{\mathrm{CoF}}, \end{array}$$

where η denotes the learning rate for the entropy estimation networks.

Similarly, at the body–tail interface, the server first performs the forward propagation to estimate the entropy $H_{i}^{t}$ according to Equations (5)–(7). Then, the parameters ${\mathrm{ha}}_{st,i}^{t-1}$ and ${\mathrm{hs}}_{st,i}^{t-1}$ are optimized to minimize the total loss $L_{\mathrm{CoF}}$, ensuring accurate modeling of the feature distribution:

(19) $$\begin{array}{cc} {\mathrm{ha}}_{st,i}^{t} & \leftarrow {\mathrm{ha}}_{st,i}^{t-1}-\eta \nabla_{{\mathrm{ha}}_{st,i}}L_{\mathrm{CoF}}, \end{array}$$

(20) $$\begin{array}{cc} {\mathrm{hs}}_{st,i}^{t} & \leftarrow {\mathrm{hs}}_{st,i}^{t-1}-\eta \nabla_{{\mathrm{hs}}_{st,i}}L_{\mathrm{CoF}}. \end{array}$$

By continuously updating these parameters, the entropy estimation mechanism adapts to the dynamic feature representations driven by the joint optimization objective.

This phase constitutes the core of the optimization process, where the entropy constraints are fully activated (i.e., $\lambda_{h},\lambda_{t}>0$). Unlike the warm-up phase which focuses solely on distortion, this phase employs a joint optimization strategy to simultaneously minimize both the reconstruction distortion and the feature transmission rate. In this stage, the gradients derived from the total loss $L_{\mathrm{CoF}}$ are back-propagated through the entire framework.

(c) Global model aggregation: To leverage collaborative knowledge, the global model aggregation phase is executed at the Fed server every *I* training rounds, corresponding to steps c1–c3 in Figure 2. First, the clients and the edge server upload their respective sub-models, including the head network $w_{ch}$, the tail network $w_{ct}$, and the server-side personalized body network $\theta_{m}$. Subsequently, the Fed server performs uniform averaging to synchronize these components:

(21) $$\begin{array}{cc} w_{ch}^{t} & =\frac{1}{N}\sum_{i=1}^{N}w_{ch,i}^{t}, \end{array}$$

(22) $$\begin{array}{cc} w_{ct}^{t} & =\frac{1}{N}\sum_{i=1}^{N}w_{ct,i}^{t}, \end{array}$$

(23) $$\begin{array}{cc} \theta_{m}^{t} & =\frac{1}{N}\sum_{i=1}^{N}\theta_{m,i}^{t}. \end{array}$$

After aggregation, the synchronized parameters are distributed back to the participants for the next round of training.

We adopt a collaborative client-server strategy to train the CoF U-SFL framework. Specifically, clients are responsible for executing the head and tail networks utilizing their local private data. Conversely, the server orchestrates the operations of the body network and the entropy estimation modules, while also managing the global model aggregation. The comprehensive workflow is outlined in Algorithm 1.
**Algorithm 1** The training process of CoF U-SFL**Require:** *b*, γ, *E*, N, and D.**Ensure:** $w^{*}$.  1:**Initialization:** $w^{0}$, $w_{i}^{0}\leftarrow w^{0}$, $I^{0}\leftarrow 1$, τ←0 and ρ←0.  2:**for** $t=\rho +1,\rho +2,\ldots ,\rho +I^{\tau }$ **do**  3:     /** Runs on clients **/  4:     **for** each client in parallel **do**  5:         $a_{h,i}^{t}\leftarrow H\left(x_{i}^{t};w_{ch,i}^{t-1}\right)$  6:         Send $a_{h,i}^{t}$ to the edge server.  7:     **end for**  8:     /** Runs on edge server **/  9:     $a_{t,i}^{t}\leftarrow B\left(a_{h,i}^{t};w_{sb,i}^{t-1}\right)$10:     Send $a_{t,i}^{t}$ to corresponding clients.11:     FP of entropy estimation network.12:     Obtain the feature entropy $H_{i}^{h}$ and $H_{i}^{t}$ using Equations (4) and (7).13:     /** Runs on clients **/14:     **for** each client in parallel **do**15:         ${\hat{x}}_{i}^{t}\leftarrow T\left(a_{t,i}^{t};w_{ct,i}^{t-1}\right)$16:         Calculate loss function value:17:         $L_{\mathrm{CoF}}=d\left(x,x^{\prime }\right)+\lambda_{h}H_{i}^{h}+\lambda_{t}H_{i}^{t}$18:         BP and send gradients to the edge server.19:     **end for**20:     /** Runs on edge server **/21:     $\theta_{sb,i}^{t}\leftarrow \theta_{sb,i}^{t-1}-\gamma \nabla_{\theta_{sb}}F_{i}\left(\theta_{sb,i}^{t-1};\xi_{i}^{t}\right)$22:     **for** each client in parallel **do**23:         $\theta_{m,i}^{t}\leftarrow \theta_{m,i}^{t-1}-\gamma \nabla_{\theta_{m}}F_{i}\left(\theta_{m,i}^{t-1};\xi_{i}^{t}\right)$24:         Send gradients to corresponding clients.25:         BP of entropy estimation network.26:   **end for**27:   /** Runs on clients **/28:   **for** each client in parallel **do**29:         $w_{ch,i}^{t}\leftarrow w_{ch,i}^{t-1}-\gamma \nabla_{w_{ch}}F_{i}\left(w_{ch,i}^{t-1};\xi_{i}^{t}\right)$30:     **end for**31:**end for**32:**Runs on the Fed server:**33:**if** $\left(t-\rho \right)\;\mathrm{mod}\;I^{\tau }=0$ **then**34:     $w_{ch}^{t}\leftarrow \frac{1}{N}\sum_{i=1}^{N}w_{ch,i}^{t}$35:     $w_{ct}^{t}\leftarrow \frac{1}{N}\sum_{i=1}^{N}w_{ct,i}^{t}$36:     $\theta_{m}^{t}\leftarrow \frac{1}{N}\sum_{i=1}^{N}\theta_{m,i}^{t}$37:     $w_{ch,i}^{t}\leftarrow w_{ch}^{t}$38:     $w_{ct,i}^{t}\leftarrow w_{ct}^{t}$39:     $\theta_{m,i}^{t}\leftarrow \theta_{m}^{t}$40:     Update $\rho \leftarrow \rho +I^{\tau }$.41:     Update τ←τ+1.42:**end if**

## 5. Performance Evaluation

We evaluate the proposed CoF U-SFL framework across two dimensions: communication efficiency and reconstruction fidelity. Communication efficiency is quantified by the total volume of feature data transmitted between the client and server during training, reflecting the reduction in overhead achieved through feature compression. Reconstruction fidelity is assessed using the peak signal-to-noise ratio (PSNR) on test datasets, ensuring that reduced communication does not compromise image quality.

### 5.1. Implementation Details

We adopt the SOTA Wireless Image Transmission Transformer (WITT) [33] as the backbone. Specifically, this backbone has a total model size of approximately 107.43 MB (with 53.72 MB for the encoder and 53.71 MB for the decoder). To align with the U-SFL topology, we partitioned the architecture into the head, body, and tail networks.

For the feature entropy estimation module, we construct a dual-layer Transformer-based hyperprior network to capture global dependencies in the feature distribution. This module introduces an additional parameter overhead of 58.69 MB.

Furthermore, the intermediate feature sizes and model allocations vary depending on the selected split points within the U-shaped architecture. Specifically, partitioning at the first and second split points yields intermediate feature dimensions of [1, 16,384, 128] (8 MB data volume) and [1,4096,192] (3 MB data volume), corresponding to client-side head network sizes of 3.05 MB and 10.61 MB, respectively.

All models were implemented in Python 3.7 using PyTorch 1.9.1 and trained on an NVIDIA GeForce RTX 4090 GPU. We used the Adam optimizer with an initial learning rate of $1\times 10^{-4}$ and a mini-batch size of 16. To ensure stable convergence, we employed a two-stage training strategy. In the initial warm-up phase, we set the entropy weights $\lambda_{h}=\lambda_{t}=0$ to optimize solely for distortion. Once stabilized, we activated the entropy constraints. Since the estimated entropy magnitude is significantly larger than the distortion loss, we empirically set $\lambda_{h}$ and $\lambda_{t}$ to the order of $10^{-5}$ to balance the gradient scales. The training process spanned 70 epochs, with uplink and downlink transmission emulated within a JSCC framework at a fixed SNR of 15 dB.

We used the DIV2K dataset [34] for training and the Kodak [35] and CLIC2021 [36] test sets for evaluation. For training, random cropping was applied to generate 256×256 patches.

### 5.2. Performance Evaluation and Analysis

We compare the proposed CoF U-SFL framework against several representative baselines as follows:

- **CL:** This method represents the ideal scenario where all client data are collected and trained on a central server using a unified WITT model. It establishes the performance upper bound for reconstruction quality, although it fundamentally violates data privacy constraints.
- **FL:** We employ the standard FedAvg algorithm, where clients train complete WITT models locally and the server aggregates model weights every 6 local epochs. This represents the conventional privacy-preserving approach that relies on full client-side computation.
- **U-SFL (WITT):** This baseline implements the standard U-SFL framework with the SOTA WITT (built on the Swin Transformer architecture) [33]. For a fair comparison, it maintains strictly consistent experimental settings with our CoF U-SFL, including the identical WITT-based network architecture, specific split points, and global aggregation frequency. This baseline provides a direct comparison to isolate and quantify the performance gains attributed to our proposed method.
- **U-SFL-FCA:** In this baseline, we implement the feature compression via an autoencoder (FCA) mechanism [19,26] within the U-SFL framework. This method compresses features at the split point through dimensionality reduction. While this approach effectively reduces communication overhead, the inevitable feature distortion introduced during the compression process results in irreversible information loss.

It is worth noting that in the aforementioned U-SFL and CoF U-SFL, the transmission over physical channels inherently provides an implicit regularization effect, and the channel noise acts as a stochastic constraint that prevents the network from overfitting to ideal communication links. Consequently, during back-propagation, the gradients derived from the noise-corrupted features force the model to discard noise-sensitive details, naturally driving the extraction of channel-robust feature representations.

As summarized in Table 1, CL naturally establishes the performance upper bound. The communication overhead of FL is highly sensitive to aggregation frequency. Aggregating every epoch results in a volume of 7.5 GB, whereas extending the interval to 6 epochs reduces this to 1.18 GB with a marginal PSNR drop of approximately 0.1 dB. However, FL imposes a high computational load on clients, requiring full forward and backward propagation of the WITT model. In contrast, standard U-SFL offloads the majority of computational burdens to the server, requiring clients to execute only the lightweight head and tail components. Specifically, when the split point is set at the first layer, the client-side model size is drastically reduced from 107.43 MB (in FL) to merely 6 MB. While this successfully redistributes computational pressure, the fundamental limitation of this framework lies in the transmission mechanism: the iterative bidirectional transfer of high-dimensional features and gradients generates excessive communication overhead. With feature sizes reaching up to 8 MB, the communication volume for features can escalate to 13.67 GB, and the total communication volume is 28.52 GB, creating a critical bottleneck that hinders practical deployment in bandwidth-limited scenarios.

As shown in Table 1, the proposed CoF U-SFL framework introduces a compact feature learning mechanism that maintains nearly identical rate-distortion performance to the vanilla U-SFL while significantly improving communication efficiency. Specifically, feature communication volume drops from 13.67 GB to 0.12 GB, a 104.6× reduction. Correspondingly, total communication decreases from 28.52 GB to 14.97 GB. This approach mitigates the communication bottleneck while preserving the low client-side computational load and privacy benefits of U-SFL, making it a viable solution for resource-constrained edge environments.

Additionally, U-SFL-FCA employs an autoencoder to compress the intermediate features. Although this strategy reduces the feature communication volume to 6.84 GB, the experimental results exhibit a trade-off in representation capability. Unlike our entropy-guided approach, the fixed compression mechanism in U-SFL-FCA lacks the adaptability to distinguish between redundancy and task-critical information. Consequently, U-SFL-FCA achieves a PSNR of only 29.3 dB compared to 30.1 dB for our method.

To investigate the mechanism behind this communication efficiency, we analyzed the entropy of features at the split point. Feature entropy serves as a fundamental metric for information redundancy, providing a theoretical basis for the efficiency gains. As shown in Table 1, by systematically comparing the feature entropy values of different methods, we reveal the essential reason behind the communication optimization achieved by the proposed CoF U-SFL. Experimental results indicate that CoF U-SFL reduces feature entropy by two orders of magnitude, from approximately 1360 in vanilla U-SFL to about 13. This proves that our proposed compact feature learning mechanism effectively eliminates information redundancy in the feature representation, laying a theoretical foundation for the improvement in communication efficiency. The entropy estimation network effectively guides the model to eliminate redundancy and learn a feature space with higher information density.

As shown in Table 2, we comprehensively evaluated the proposed CoF U-SFL against the vanilla U-SFL baseline across three datasets with varying resolutions: CLIC 2021 (high), MS-COCO (medium), and CIFAR-10 (extremely small). The experimental results clearly demonstrate that our method is highly effective across different spatial scales, successfully achieving massive reductions in both feature volume and feature entropy while maintaining comparable image reconstruction quality (PSNR). Specifically, for the CLIC 2021 and MS-COCO datasets, since the input images are uniformly processed into 256×256 patches in our experimental setup, the intermediate feature volumes generated by the vanilla U-SFL are inherently identical (13.67 GB). By applying our feature entropy estimation mechanism, CoF U-SFL drastically compresses these volumes down to 0.12 GB and 0.22 GB, respectively. Furthermore, for extremely small-scale images like CIFAR-10 (32×32), the intrinsic data dimension is much smaller than the standard patch size, leading to a naturally smaller baseline feature volume (1.71 GB for U-SFL). Because such low-resolution images inherently contain less spatial redundancy, the theoretical room for compression is relatively restricted compared to high-resolution datasets. However, the results confirm that even under these constrained conditions, CoF U-SFL still effectively compresses the feature volume to 0.057 GB and drops the feature entropy from 1340 to 45.

**Figure 4:**
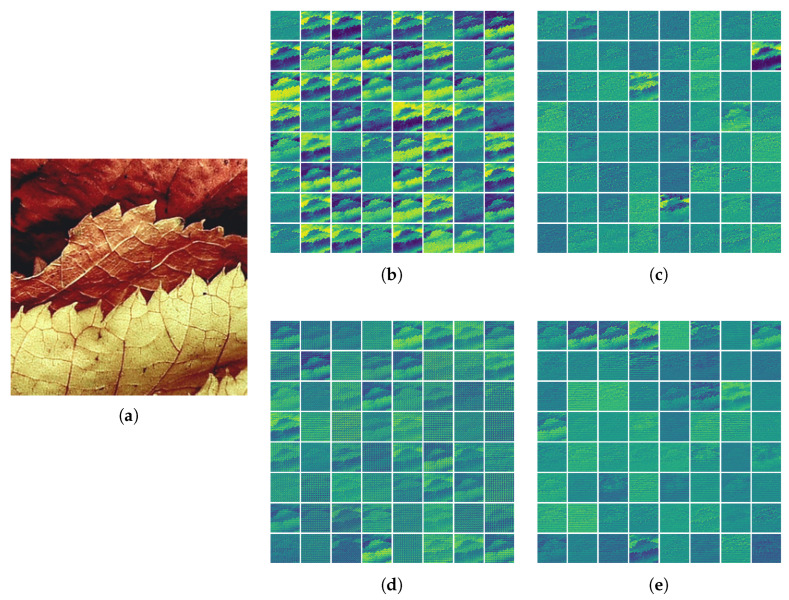
Visual comparison of feature maps. (**a**) The original input image. (**b**) Feature map of U-SFL (WITT) at the head–body interface. (**c**) Feature map of CoF U-SFL at the head–body interface. (**d**) Feature map of U-SFL (WITT) at the body–tail interface. (**e**) Feature map of CoF U-SFL at the body–tail interface. Compared to the contour-heavy U-SFL (WITT) features (**b**,**d**), the CoF U-SFL features (**c**,**e**) exhibit highly abstract patterns, indicating reduced spatial redundancy.

As illustrated in Figure 4, part (a) displays the original input image. The feature maps generated by the vanilla U-SFL (b,d) preserve distinct spatial structures and perceptible contours, indicating strong spatial correlation at both split interfaces. In contrast, CoF U-SFL features (c,e) manifest as highly abstract patterns where geometric details are significantly obfuscated. This suppression of spatial distinctness provides intuitive evidence that our method successfully reduces non-task-critical visual redundancy. Consequently, it encodes the data into a compact, information-dense latent representation, thereby effectively minimizing the feature entropy required for transmission.

To further evaluate the robustness and adaptability of the proposed CoF U-SFL framework, we further investigated the impact of split-point selection. Specifically, we conducted comparative experiments using two representative split points defined within the WITT architecture:

- **Split Point 1 (Shallow Split):** Positioned after the first Transformer encoder block. As detailed in IV.A, while the client-side model is lightweight, the feature maps at this shallow stage preserve rich low-level spatial details and high dimensionality. Consequently, this results in greater communication overhead.
- **Split Point 2 (Mid Split):** Positioned after the second Transformer encoder block. The features have undergone deeper transformations, capturing more abstract information with reduced spatial resolution. This significantly alleviates the communication overhead, albeit at the cost of deploying a larger head network on the edge client.

Table 3 presents a comparative analysis of performance and communication efficiency across different split strategies on the Kodak dataset. To ensure a fair comparison and isolate the impact of the split point, the server aggregation interval was fixed at every 6 rounds for all experiments. First, regarding the vanilla U-SFL, the communication overhead is strictly dictated by the split depth. The shallower split point 1 outputs high-dimensional feature maps, inevitably resulting in a much heavier transmission load. As the network deepens to split point 2, the intrinsic feature entropy naturally decreases from 1360 to 463, causing the total communication volume to drop from 28.52 GB to 12.04 GB.

Crucially, CoF U-SFL exhibits a distinct trend where higher initial redundancy correlates with greater compression gains. At split point 1, where intermediate features possess high dimensionality and redundancy, our method achieves its most significant efficiency improvement. Specifically, by reducing feature entropy from 1360 to 13, the feature communication volume drops from 13.67 GB to 0.12 GB, achieving a 104.6× compression ratio. Similarly, at split point 2, where the vanilla feature volume decreases to 5.12 GB, our mechanism further compresses the data to 0.10 GB. This comparison demonstrates the generality of our approach. Regardless of the initial feature dimension at the split point, CoF U-SFL effectively guides the model to learn a compact, information-dense feature representation. In particular, the superior compression capability at shallow network layers makes our method especially suitable for resource-constrained edge devices facing prohibitive transmission costs.

Furthermore, regarding model performance, CoF U-SFL matches the PSNR values of vanilla U-SFL at both split points. This confirms that our communication optimization is achieved by eliminating informational redundancy within the features rather than at the expense of accuracy. Notably, the reconstruction performance at split point 1 is marginally higher than at split point 2, which is attributed to the server undertaking a heavier computational load. This finding highlights the inherent trade-off between client-side computational cost and model performance, allowing for flexible selection based on available resources. Consequently, these results provide strong evidence that the CoF U-SFL framework is not a specialized optimization tailored to a specific model structure, but rather a general solution for enhancing communication efficiency. It can flexibly adapt to diverse system design requirements, whether the priority lies in alleviating client-side computational burden or minimizing communication overhead.

## 6. Conclusions

We proposed CoF U-SFL, a collaborative image coding framework designed for U-shaped split federated learning. By integrating a dual-layer Transformer-based hyperprior network, CoF U-SFL models the statistical distribution of split-layer features to estimate entropy without accessing raw data. We established a stable end-to-end training pipeline through a joint optimization objective that balances entropy constraints with distortion metrics. Experimental results indicate that CoF U-SFL reduces feature communication overhead by 104.6× while maintaining comparable image reconstruction quality. This approach effectively balances privacy preservation, communication efficiency, and image fidelity within distributed learning environments. Future research will investigate defenses against reconstruction attacks and extend the framework to video coding applications in high-concurrency edge systems.

## Figures and Tables

**Table 1:** Comparison of performance and communication overhead across different distributed learning methods on the CLIC 2021 test set.

Method	Aggregation	PSNR (dB)	Feature Vol. (GB)	Total Comm. (GB)	Entropy of Feature
CL	–	31.0	–	–	–
FL (Every Round)	Every Round	30.6	–	7.50	–
FL (6 Rounds)	6 Rounds	30.5	–	1.18	–
U-SFL (WITT)	6 Rounds	30.1	13.67	28.52	1360
U-SFL-FCA	6 Rounds	29.3	**6.84**	21.69	–
**CoF U-SFL (Ours)**	6 Rounds	**30.1**	**0.12**	**14.97**	**13**

**Table 2:** Comparison of PSNR, feature volume, and feature entropy across datasets of varying resolutions.

Dataset	Method	PSNR (dB)	Feature Vol. (GB)	Entropy of Feature
CLIC 2021	U-SFL (WITT)	30.1	13.67	1360
	CoF U-SFL (ours)	30.1	0.12	13
MS-COCO	U-SFL (WITT)	31.2	13.67	1340
	CoF U-SFL (ours)	31.1	0.22	22
CIFAR-10	U-SFL (WITT)	29.8	1.71	1340
	CoF U-SFL (ours)	29.9	0.0574	45

**Table 3:** Performance and efficiency comparison under different model split points.

Split Point	Method	PSNR (dB)	Communication (GB)	Feature Vol. (GB)	Feature Entropy	Compression Ratio of Feature
Split Point 1	U-SFL (WITT)	30.1	28.52	13.67	1360	1.0x
	CoF U-SFL (Ours)	**30.2**	**14.97**	**0.12**	**13**	**104.6x**
Split Point 2	U-SFL (WITT)	30.3	12.04	5.12	463	1.0x
	CoF U-SFL (Ours)	**30.3**	**7.02**	**0.10**	**9**	**51.4x**

## Data Availability

The original contributions presented in this study are included in the article. Further inquiries can be directed to the corresponding author.
